# Supplementary material for: Factors associated with retention in Quitline counseling for smoking cessation among HIV-positive smokers receiving care at HIV outpatient clinics in Vietnam
Source: PLoS One. 2024 Dec 31;19(12):e0316250. doi: 10.1371/journal.pone.0316250 (PMC11687760; doi:10.1371/journal.pone.0316250)
Supplement: Attachment — Submitted filename: Minor revisions PLOS One Article.docx [file pone.0316250.s002.docx]

Minor revisions: Grammatical errors

1. Material and methods

Data source

5^th^ paragraph – 2^nd^ line – Vietnamese language (the word language is missing)

5^th^ paragrapgh – 5^th^ line – All participants were provided……. (the word ‘were’, is missing)

2. Results

Socio-demographic characteristics

1^st^ paragraph – 1^st^ line – Most participants were males (in plural)

3. Discussion

4^th^ paragraph – 4^th^ line – rates (in plural)

Besides that, well written study.
